# TaqMan quantitative real-time PCR for detecting *Avipoxvirus* DNA in various sample types from hummingbirds

**DOI:** 10.1371/journal.pone.0230701

**Published:** 2020-06-11

**Authors:** Hanna E. Baek, Ruta R. Bandivadekar, Pranav Pandit, Michelle Mah, Ravinder N. M. Sehgal, Lisa A. Tell

**Affiliations:** 1 Department of Biology, San Francisco State University, San Francisco, CA, United States of America; 2 Department of Medicine and Epidemiology, School of Veterinary Medicine, University of California, Davis, CA, United States of America; 3 EpiCenter for Disease Dynamics, One Health Institute, School of Veterinary Medicine, University of California, Davis, CA, United States of America; University of Helsinki, FINLAND

## Abstract

**Background:**

Avian pox is a viral disease documented in a wide range of bird species. Disease-related detrimental effects can cause dyspnea and dysphagia, and birds with high metabolic requirements, such as hummingbirds, are thus especially vulnerable to the pathogen. Hummingbirds have a strong presence in California, especially in urban environments. However, little is understood regarding the impact of pox virus on hummingbird populations. Currently, diagnosing a pox infection relies on obtaining a tissue biopsy, which poses significant risks to birds and challenges in the field. Understanding the ecology of hummingbird pox viral infections could be advanced by a minimally invasive ante-mortem diagnostic method. Our aim was to address whether pox infections can be diagnosed using integumentary system samples besides tissue biopsies. To meet this goal, we tested multiple integumentary sample types using a quantitative real-time PCR assay. A secondary study goal was to determine which sample types (ranging from minimally to highly invasive sampling) were optimal for identifying infected birds.

**Methodology and principal findings:**

Pox-like lesion tissue, pectoral muscle, feathers, toenail clippings, blood, and swabs (both pox-like lesion tissue and non pox-like lesion tissue) were taken from live birds and carcasses of two species of hummingbirds found in California. To maximize successful diagnosis, especially for samples with low viral load, a real-time quantitative PCR assay was developed for detecting the hummingbird-specific *Avipoxvirus* 4b core protein gene. *Avipoxvirus* DNA was successfully amplified from all sample types obtained from 27 individuals. These results were compared to those of conventional PCR and comparisons were also made among sample types, utilizing lesion tissue samples as the gold standard.

**Conclusions and significance:**

Hummingbird avian pox can be diagnosed without relying on tissue biopsies. We identify that feather samples, of which contour feathers yielded the best results, can be used for diagnosing infected birds, thus reducing sampling risk. In sum, the real-time PCR assay detected viral DNA in various integumentary system sample types and will be useful in future studies of hummingbird disease ecology.

## Introduction

Avian pox is a disease caused by strains of the genus *Avipoxvirus* and can manifest in a cutaneous (dry) and a diphtheritic (wet) form [1]. With the dry form of pox, wart-like growths form primarily on non-feathered body regions and are relatively easy to detect. In contrast, the wet form of a pox infection is more difficult to visually detect in a free-ranging bird, as it is characterized by growths on the mucosal membranes in the mouth, esophagus, and lungs [2]. Both forms of pox can cause respiratory or alimentary tract compromise that can ultimately lead to mortality of infected birds [3]. Avian pox can infect a wide range of bird species, including hummingbirds [4]. Hummingbirds appear to be especially vulnerable to the effects of the pox virus because of their high metabolic requirements [5]. Understanding how avian pox impacts hummingbird populations is important since these iconic pollinators can be indicators of environmental health. As such, information about hummingbird diseases can inform us about the general health of the environments they inhabit [5].

Avian pox has traditionally been diagnosed by histological analyses [1,4] and electron microscopy [6] of tissue samples from lesions found on skin surfaces. Though histological analysis is a reliable method for pox diagnosis, it is difficult to do on frozen samples and Bollinger bodies, intracytoplasmic inclusion bodies found in the tissues of pox infected birds [7], are not always apparent [8]. Additionally, histology requires a tissue biopsy. Taking tissue samples of lesions for analyses is a reliable method for detecting avian pox; however, the procedure presents several challenges. Obtaining tissue biopsies requires anesthesia and presents the risk of significant hemorrhage or open wounds. Thus, it is important to consider alternative methods for diagnosing avian pox in live birds.

Since avian pox is a disease that primarily targets the integumentary system [9], we hypothesized that it would be possible to detect avian pox in other sample types that are components of the integumentary system. Taking different sample types would allow for minimal animal harm, especially in the case of field sampling; however, a method for testing the different samples must be developed for reliable diagnosis of avian pox infection.

Polymerase chain reaction (PCR) testing has been used to detect many avian pathogens, such as avian malaria [10,11,12] and avian infectious bronchitis [13]. PCR targets and amplifies specific regions of the pathogen’s genome, which may allow for detection even when the disease is asymptomatic or cannot be diagnosed using histopathology [10] or viral isolation [14]. Conventional PCR has been used to diagnose avian pox infections, and is straightforward since avian pox is a DNA virus [15]. A PCR protocol has been developed, which amplifies a 578-bp fragment of fowlpox virus (FPV) from skin tissue samples and respiratory swabs taken from chickens showing signs of pox infection [2]. A multiplex PCR protocol that could detect both *Avipoxvirus* and papillomavirus infections has also been described [16]. Using superficial skin swabs from birds in the field and of preserved museum skin specimens that demonstrated symptoms of viral infection, it was reported that detection of multiple strains of both *Avipoxvirus* and papillomavirus is possible through PCR [16]. This protocol was also used in another study where, in addition to superficial skin swabs, blood and tissue samples taken from symptomatic wild birds were tested and it was found that swab and tissues samples generated significantly more avian pox positives than blood samples [17].

In addition to conventional PCR, another method for diagnosing avian pox infections is quantitative real-time PCR. Real-time PCR allows for a quantitative analysis of pathogens without the need for additional diagnostic tests, such as gel electrophoresis for conventional PCR. As a complementary method to conventional PCR, a real-time PCR protocol was developed to detect avian pox in archived blood samples from Hawai’i Amakihi [18]. Cases were confirmed either from the successful culturing of *Avipoxvirus* or through conventional PCR testing. The protocol demonstrated that real-time PCR could be used to positively identify avian pox infections and estimate viral load [18]. As such, real-time PCR may be a useful tool for detecting avian pox infection in hummingbirds as it is a more sensitive assay for detecting viral particles and can be used to detect pox viral DNA in non-lesion tissue samples, which are likely to have lower viral loads.

Although it has been shown that avian pox can be detected via conventional PCR [2,16,17] and real-time PCR [18] in other bird species, we lacked a real-time PCR method for detecting the specific strain of *Avipoxvirus* found in hummingbirds. The protocol published for Hawai’i honeycreepers employed a real-time PCR protocol that amplifies a segment of the *Avipoxvirus* 4b core protein gene [18]. They successfully amplified *Avipoxvirus* DNA in several of their samples, but in their analyses, the Hawaiian strain clusters with canarypox [19], which is distinct from the strain of pox found in hummingbirds [4]. Our previous work in hummingbirds used a conventional PCR protocol that amplified a segment of the 4b core protein gene of FPV, which allowed for confirmation of avian pox infection [4]. That work confirmed that hummingbirds are infected with a strain not found in other surveyed bird species; affirming a study [20] that found that poxviruses infecting different species of birds demonstrated considerable variation. It was determined that the pox virus found in hummingbirds seems to cluster with pox viruses isolated from avian species for which a specific diagnostic PCR protocol has not been developed [4]. Thus, in order to most accurately screen hummingbird for pox infection, a protocol that is specific to the strain of avian pox found in hummingbirds would be beneficial.

Developing an accurate method for detecting pox infection without taking tissue biopsies would allow for field studies of *Avipoxvirus* infections. By analyzing and comparing the results from different sample types using real-time PCR as a complement to conventional PCR, it can be determined which sample type might be optimal for screening for pox infections and would allow researchers to prioritize sample collection when in the field or laboratory.

The goals of this study were to 1) determine if pox infection could be diagnosed without a tissue biopsy and 2) determine which integumentary system sample types allow for optimal screening of hummingbirds for pox infection. We describe the development of a quantitative real-time PCR protocol for detecting *Avipoxvirus* in a variety of sample types taken from Anna’s Hummingbirds (*Calypte anna*; ANHU) and a *Selasphorus* spp. Hummingbird (SEHU) to determine if avian pox could be diagnosed without a tissue biopsy. We determined the effectiveness of the real-time PCR by comparing results to those from conventional PCR. Finally, assessments of the results from different sample types, in comparison to lesion tissue samples, were made to determine the least invasive sample type. Our results suggest that sampling contour feathers appears to be the least invasive means of detecting positive pox virus infections in hummingbirds.

## Materials and methods

We received approval by the United States Fish and Wildlife Service (Permit: MB55944B-2), United States Geological Survey Bird Banding Laboratory (Permit: 23947), California Department of Fish and Wildlife (Permit: SC-013066), and the UC Davis Institutional Animal Care and Use Committee (Protocol: 20355) to conduct all research within the scope of this study.

### Sample collection

**Fig 1:**
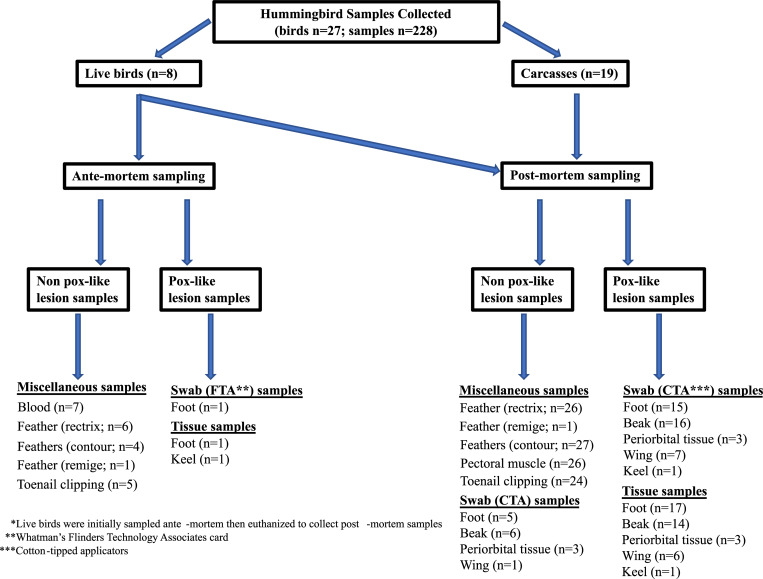
Schematic of sample collection from hummingbirds (n = 27) with lesions that were visually consistent with pox viral infections. Both carcasses (n = 19 birds) and field-caught individuals (n = 8 birds) were included in the study.

Samples (n = 228) from 27 hummingbirds (ANHU n = 26; SEHU n = 1) with lesions that were visually consistent with pox viral infections were collected (Fig 1). Due to missing feathers, the *Selasphorus* spp. bird could only be identified to genus. Of the 27 hummingbirds, some were carcasses (n = 19 birds) that were collected from California rehabilitation centers where birds did not survive the rehabilitation process. Some birds (n = 8 birds) were euthanized during live sampling since they were considered unfit for survival owing to heavy pox infections.

To compare samples taken ante- and post-mortem, feather (contour and rectrices), toenail clip, and pox-like lesion tissue samples were collected while birds (n = 6) were alive and similar samples were collected post-mortem (S1 Table). This step was taken in order to address the issue of possible sample contamination caused by viral particles that could have dislodged while the carcasses were in storage. Blood samples (n = 7 birds) were taken via clipping the distal 10% of the toenail and collected onto Whatman FTA paper (GE Healthcare, Chicago, Illinois, USA) [21]. A swab of pox-like lesion tissue was collected from the foot of a live bird using FTA paper [21].

Samples collected from carcasses included feather, toenail clippings, pox-like lesion tissue, and pectoral muscle tissue. In addition, swabs of tissues with pox-like lesions and lacking pox-like lesions were taken. Although pectoral muscle is not considered as part of the integumentary system, it was included in this study because it may be an optimal sample type for assessing pox infection in specimens destined to be study skins. For toenail clip samples, approximately 10% of the toenail was sampled from the carcasses. Tissue samples were taken from pox-like lesions on the wings, feet, periorbital region, or beak. For birds with pox-like lesions on multiple regions, tissue samples were taken from each region using a sterile no. 15 scalpel blade. Pox-like lesion swabs were obtained using sterile cotton-tipped applicator (CTA) swabs soaked in saline solution and pectoral muscle tissue (0.2–0.4 cm diameter) was taken from each carcass using a dermal punch (Miltex Inc., York, Pennsylvania, USA, catalog #s MLTX33-31 and MLTX33-34, respectively). Differing sample types and number of samples were taken from birds based on presence of lesions that were visually consistent with pox viral infections. Several different sample types were taken from each individual to increase the likelihood of detecting pox viral DNA as well as to determine which sample types could be reliably used to test for pox infections.

### Samples and DNA extraction

For the initial TaqMan protocol development, only contour feather and pox-like lesion tissue samples were extracted. These extracted samples were used for the initial protocol development, but sample results were not included in the data analysis presented in this manuscript. Approximately 5 mg of pox-like lesion tissue or five feathers was added to a 96-well deep-well grinding block (Greiner Bio-One, Monroe, North Carolina, USA, cat #780215) with 600 μl of ATL Buffer, 60 μl of Proteinase K (Qiagen, Valencia, California, USA), and two stainless-steel beads (Fisher Scientific, Waltham, Massachusetts, USA, cat #02-215-512). The grinding block was sealed, then the samples were pulverized in a 2010 Geno/Grinder homogenizer (SPEX SamplePrep, Metuchen, New Jersey, USA) at 1,750 rpm for 2.5 min. Lysate was incubated for 15 min at 56° C. 200 μl of lysate was removed and used for total nucleic acid (TNA) extraction. TNA extraction was performed on a semi-automated extraction system (QIAamp 96 DNA QIAcube HT Kit, QIAcube; Qiagen, Valencia, California, USA) according to manufacturer’s instructions and eluted in 100 μl of diethylpyrocarbonate (DEPC)-treated water. These extraction protocols were modified for sample analysis as detailed later.

Once the TaqMan protocol was developed and validated, DNA was extracted from 228 samples collected from 27 individual hummingbirds. For DNA extraction of blood samples, a small piece of Whatman FTA paper (GE Healthcare, Chicago, Illinois, USA) with blood was cut using sterilized dissection scissors (Thermo Fisher Scientific, Carlsbad, California, USA; Stainless steel). The same method was used for DNA extraction of the FTA swab of pox-like lesion tissue. For rectrices and remiges, DNA was extracted from feather sheath bases, which were cut using sterilized dissection scissors (Thermo Fisher Scientific, Carlsbad, California, USA; Stainless steel). DNA was also extracted from four whole contour feathers taken from the pectoral region and from toenail clippings. DNA was extracted from each sample taken from captured birds and carcasses using the Wizard Genomic DNA Purification kit (Promega Corp., Madison, Wisconsin, USA) according to the manufacturer’s instructions and eluted in 125 μl of DEPC-treated water. All samples taken from carcasses and live birds were stored at -80° C for up to one year before DNA was extracted.

Extracted DNA samples were analyzed using the Qubit 2.0 Fluorometer (Thermo Fisher Scientific, Carlsbad, California, USA) to ensure successful extraction of DNA. These extracted DNA samples were stored at -20° C until PCR analysis.

### Conventional PCR amplification of *Avipoxvirus* DNA and sequencing

Extracted DNA was tested via PCR to amplify a section of the *Avipoxvirus* 4b core protein gene, using a protocol adapted from a previously published study [4]. 4 μl of extracted DNA were used in 25 μl reactions containing 5 μl 5x green PCR buffer, 2.5 μl MgCl_2_ (1.0–4.0mM), 0.5 μl DNTP, 1 μl each of forward and reverse primers [4], 0.125 μl GoTaq Flexi DNA Polymerase (Promega Corp., Madison, Wisconsin, USA), and 10.875 μl Ultrapure water. The forward and reverse primers used in these reactions were those used in a previous study [4]; however, all other ingredients of the reactions were adjusted for use with GoTaq Flexi DNA Polymerase (Promega Corp., Madison, Wisconsin, USA). Reactions were run through an initial denaturing period of 95° C for 5 min, followed by 40 cycles of 95° C for 30 sec, 50° C for 30 sec, and 72° C for 1 min, and the final step of 72° C for 7 min [4]. Products were visualized on a 1.8% agarose gel. PCR products from samples that tested positive were sent to Elim Biopharmaceuticals, Inc (Hayward, California, USA) for sequencing. One PCR product from each hummingbird (n = 27) was sequenced and originated from various sample types (pox-like lesion tissue, n = 12; blood, n = 1; rectrices, n = 5; contour feathers, n = 1; pox-like lesion tissue swabs, n = 2; pectoral muscle, n = 3; toenail clippings, n = 3). PCR products selected to represent each bird were those that appeared the brightest when analyzed through gel electrophoresis, and were deemed to have the greatest likelihood of returning high quality sequences. The returned nucleotide sequences were manipulated and aligned using Geneious® version 11.1.5 (Biomatters, Inc., San Diego, California, USA), then searched through the National Center for Biotechnology Information Basic Local Alignment Search Tool (BLAST).

### Real-time PCR assay development, validation, and sample analysis

The assay was designed to target the 4b core protein gene of *Avipoxvirus* (AAPV) (GenBank accession JX418296.1). Two primers (vAAPV-124f ACGTCAACTCATGACTGGCAAT and vAAPV-246r TCTCATAACTCGAATAAGATCTTGTATCG) and an internal hydrolysis probe (vAAPV-159p-FAM-AGACGCAGACGCTATA-MGB, 5’ end, reporter dye FAM [6-carboxyfluorescein], 3’ end, quencher dye NFQMGB [Non-Fluorescent Quencher Minor Grove Binding]) were designed using Primer Express Software (Thermo Fisher Scientific, Carlsbad, California, USA). The 123 base pair amplicon was entered into BLAST (NCBI) to confirm unique detection.

In the initial development of the assay, each real-time PCR reaction contained 20X primers and probe with a final concentration of 400nM for each primer and 80nM for the probe, 7 μl of commercially available PCR master mix (TaqMan Universal PCR Master Mix, Thermo Fisher Scientific, Carlsbad, California, USA, cat #4318157) containing 10mL Tris-HCl (pH 8.3), 50mM KCl, 5mM MgCl_2_, 2.5 mM deoxynucleotide triphosphates, 0.625U AmpliTaq Gold DNA polymerase per reaction, 0.25 U AmpErase UNG per reaction, and 5 μl of diluted extracted DNA. The real-time PCR was performed using the ABI PRISM 7900 HTA FAST (Thermo Fisher Scientific, Carlsbad, California, USA). The following amplification conditions were used: 50° C for 2 min, 95° C for 10 min, 40 cycles of 95° C for 15 sec and 60° C for 1 min. Fluorescent signals were collected during the annealing phase and Cq (quantification cycle) values were extracted with a threshold of 0.2 and baseline values of 3–10. Cq values are the number of cycles required for the fluorescent signal to exceed the background level. Cq values are inverse to the copies of target DNA in a sample (i.e. lower Cq values indicated high amounts of target sequence). A no template control (DEPC-treated water) was run with all assays to ensure absence of non-specific binding of the primers and probes. A reference gene, eukaryotic 18S assay (Hs99999901_s1, Applied Biosystems, Thermo Fisher Scientific, Carlsbad, California, USA), was run with each sample to confirm successful DNA extraction and lack of PCR inhibitors. Positive controls (AAPV plasmid and pooled DNA for 18S) were run with their respective assay to ensure the assay was working properly.

**Fig 2:**
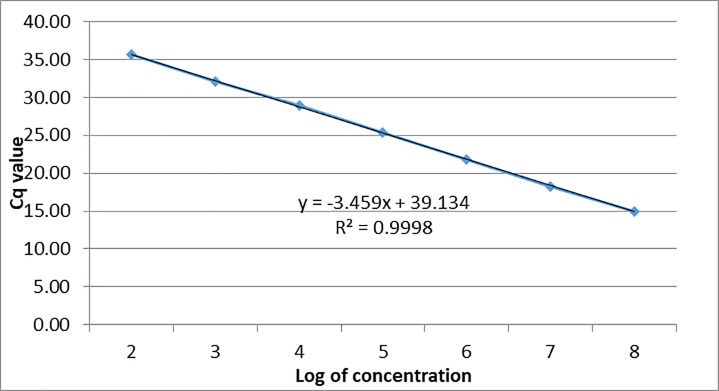
Standard curve developed for absolute quantification of viral DNA copies developed through a triplicate test of 10-fold serial dilutions of *Avipoxvirus* plasmid using the PCR master mix (TaqMan Universal PCR Master Mix, Thermo Fisher Scientific, Carlsbad, California, USA) used for assay development.

The AAPV assay was validated for efficiency and sensitivity by running a 10-fold standard curve (Fig 2) in triplicate of serial dilutions made from PCR2.1 plasmid DNA (Eurofins Genomics LLC, Louisville, Kentucky, USA) containing the AAPV amplicon. The assay was validated for real-time PCR amplification using the PCR master mix from assay development (TaqMan Universal PCR Master Mix, Thermo Fisher Scientific, Carlsbad, California, USA, cat #4318157). The real-time PCR assay was found to be 94.5% efficient and sensitive enough to detect as few as 10 copies of the target gene per real-time PCR reaction (R^2^ value = 0.9998).

**Fig 3:**
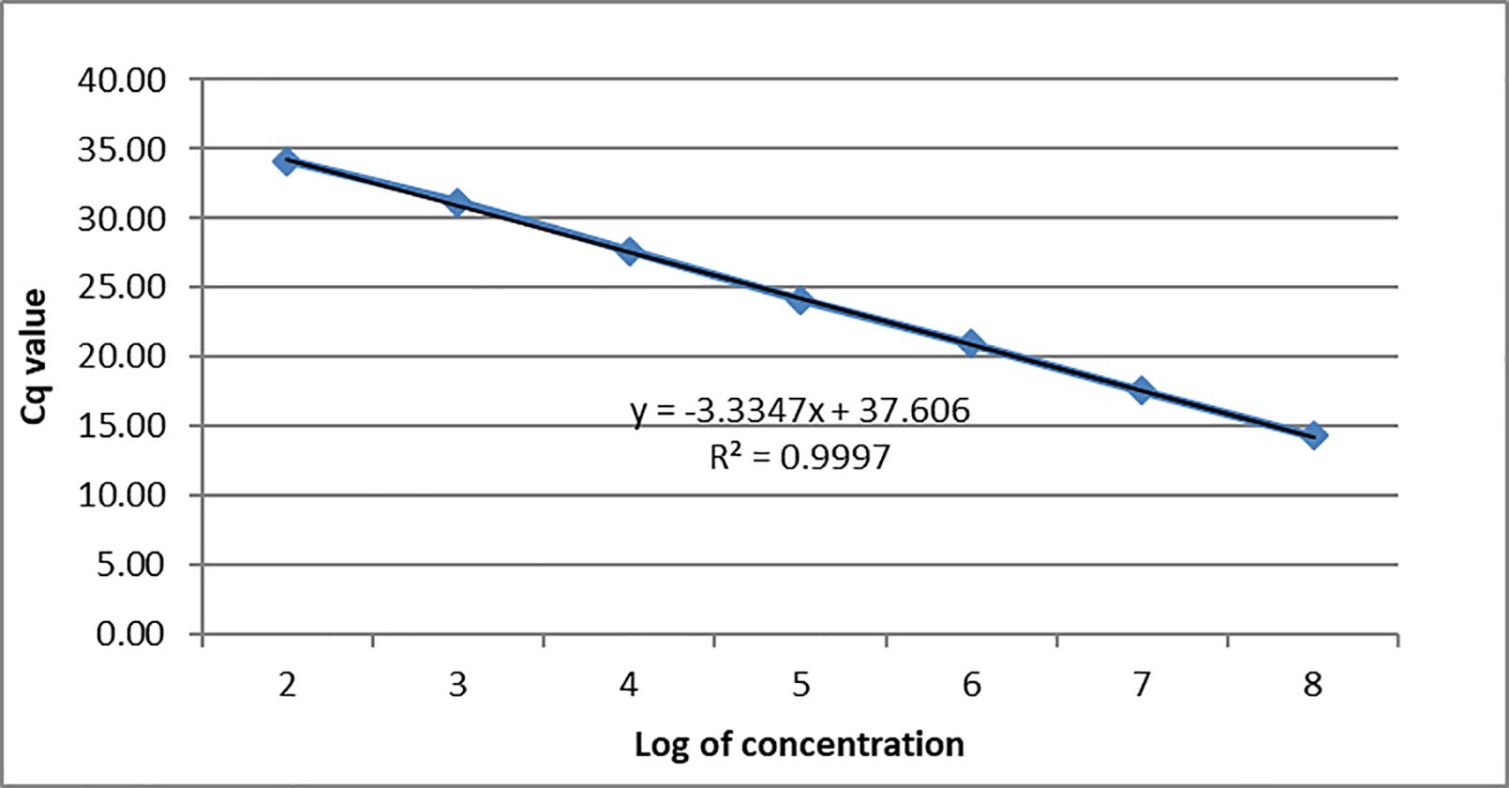
Standard curve developed for absolute quantification of viral DNA copies developed through a triplicate test of 10-fold serial dilutions of *Avipoxvirus* plasmid using the PCR master mix (Taqman Fast Advanced Master Mix, Thermo Fisher Scientific, Carlsbad, California, USA) used for sample analysis.

Samples were analyzed using a different commercially available PCR master mix (Taqman Fast Advanced Master Mix, Thermo Fisher Scientific, Carlsbad, California, USA, cat #4444557). The AAPV assay was validated for efficiency and sensitivity for use with this PCR master mix as well. The assay was validated by running a 10-fold standard curve (Fig 3) in triplicate of serial dilutions made from PCR2.1 plasmid DNA (Eurofins Genomics LLC, Louisville, Kentucky, USA) containing the AAPV amplicon. The real-time PCR assay was found to be 99.3% efficient and sensitive enough to detect as few as 10 copies of the target gene per real-time PCR reaction (R^2^ value = 0.9997).

Extracted DNA was tested via real-time PCR to amplify a section of the *Avipoxvirus* 4b core protein gene. Samples were analyzed using the validated assay with the following modifications from assay development. As stated earlier, a different commercially available PCR master mix was used (TaqMan Fast Advanced Master Mix, Thermo Fisher Scientific, Carlsbad, California, USA, cat #4444557), but the same concentrations of reagents were used (7 μl master mix: 5 μl extracted DNA). Reactions were run on a different system, CFX 96 Touch Real-Time PCR Detection System (Bio-Rad, Hercules, California, USA), under the same amplification conditions as the validated protocol: 50° C for 2 min, 95° C for 10 min, 40 cycles of 95° C for 15 sec and 60° C for 1 min. A positive control (AAPV plasmid) was run with all assays to ensure the assay was working properly; however, pooled DNA for 18S was not included in the assay during sample analysis. A no template control (purified water) was also run with all assays to ensure the absence of non-specific binding of the primers and probe. Fluorescence signals were collected during amplification and Cq values were extracted for each sample.

### Quantification of viral DNA

To establish a method for quantifying the number of copies of AAPV target genes, a plasmid standard curve was prepared in triplicate using 10-fold serial dilutions. This method was developed so that viral loads in samples from future studies will be comparable after normalizing for differences in sample weights. Even though absolute values were not calculated for samples tested in this study, we determined that future studies could use the following formula to determine absolute numbers (*abs*): Log^10^((Cq-*y*) ÷ *s*), where *y* is the y-intercept and *s* is slope obtained from a plotted standard curve (Fig 1). To determine the number of copies per well, the following formula was used: 10^*abs*^ ÷ 2. The copy number per well (1μl DNA) was divided by two since there are two copies of the gene per cell. To determine molecules/μl, the following formula was used:

$$\frac{{Avogadro}^{\prime }s\;number\;(6.02\times 10^{23}\;{mol}^{-1})\times plasmid\;concentration\;(\frac{g}{\mu l})}{Molecular\;weight\;((plasmid\;length+insert)\times 660\frac{g}{mol})}$$

### Assessment of reliability of real-time PCR in detecting positives

Cohen’s kappa statistics (*k*) was used to evaluate the agreement between conventional and real-time PCR results for each sample type. The closer the value of *k* to 1, the more the agreement between the two tests. Cohen’s *k* is prone to paradoxical results when the data is imbalanced [22]. To address this, we also report two additional supportive metrics of positive agreement and negative agreement [22]. Positive agreement estimates the conditional probability, given that one of the tests, randomly selected, gives a positive result, the other test will also do so. Similarly, the negative agreement estimates the conditional probability for negative results [22]. To understand the performance of the newly developed real-time PCR, we estimated the sensitivity, positive predictive value (PPV), and F-1 (harmonic mean of sensitivity and PPV) statistic for each sample type [23]. For this analysis, the true status (positive or negative for pox) of an individual bird was determined based on the results of all tests (both conventional and real-time PCR) performed on samples taken from the bird. By this criterion, a bird was assumed to be truly positive if any sample was detected positive for either conventional PCR or real-time PCR. The *k* statistic was also calculated to understand the agreement between different sample types for the real-time PCR.

## Results

Of 228 samples that were fluorometrically analyzed, 174 demonstrated double-stranded DNA concentrations of at least 0.5 ng/mL and the remaining 54 demonstrated concentrations lower than 0.5 ng/mL.

All 228 samples were tested using the conventional PCR assay for the presence of avian pox virus (S2 Table), of which 90% (n = 205) tested positive (Table 1). This includes the subset of samples in which similar samples were taken ante-mortem and post-mortem to address the possibility of contamination during freezer storage (S3 Table). Pox-like lesion tissue samples (n = 43/43) as well as pox-like lesion tissue swabs (n = 42/42) were most commonly positive (100%), but all other sample types tested positive as well (Table 1). 100% of remiges (n = 2/2) and FTA swabs of pox-like lesion tissue (n = 1/1) also tested positive; however, the low sample size must be considered. Toenail clip and pectoral muscle tissue samples were also positive at a high frequency, with 90% (n = 26/29) of toenail clip samples and 88% (n = 23/26) of pectoral muscle tissue samples determined as positive for pox virus using the conventional PCR assay. Contour feathers were the least likely to be positive at 77% (n = 24/31) when analyzed using the conventional PCR assay but were very likely (100%) to be positive when tested with the real time PCR assay (n = 31/31).

**Table 1:** Summary of sample types (n = number of samples; percentage) taken from Anna’s Hummingbirds (n = 26) and a *Selasphorus* spp. where *Avipoxvirus* was detected by conventional and real-time polymerase chain reaction assay.

Sample Type	n (Total number of samples)	Conventional PCR-positive for *Avipoxvirus*	Real-time PCR-positive for *Avipoxvirus*
Tissue: Pox-like Lesions	43	43 (100%)	41 (95%)
Tissue: Pectoral Muscle	26	23 (88%)	26 (100%)
Blood	7	6 (86%)	7 (100%)
Toenail Clippings	29	26 (90%)	27 (93%)
Feathers: Retrices	32	26 (81%)	28 (88%)
Feathers: Remiges	2	2 (100%)	2 (100%)
Feathers: Contour	31	24 (77%)	31 (100%)
Swab (CTA): Pox-like Lesion Tissue	42	42 (100%)	42 (100%)
Swab (CTA): Non Pox-like Lesion Tissue	15	12 (80%)	12 (80%)
Swab (FTA Card): Pox-like Lesion Tissue	1	1 (100%)	1 (100%)

CTA, Cotton-tipped applicator

PCR product sequences from 24 birds were 100% identical to the sequence for pox virus in Anna’s Hummingbirds that was previously published (GenBank accession JX418296.1) [4]. For the three remaining birds, sequences from two birds were of insufficient quality to accurately determine nucleotide differences. With the final individual Anna’s Hummingbird a distinct lineage was detected that differed from the common consensus sequence by three base pairs (GenBank accession MT332851).

All 228 samples were analyzed through the AAPV assay (S4 Table) and the average Cq values per sample type were calculated (Table 2). The real-time PCR found that 95% (n = 217) of 228 samples tested positive for *Avipoxvirus*. This includes the subset of samples in which similar samples were taken ante-mortem and post-mortem (S3 Table). Of the samples that were negative for *Avipoxvirus* (n = 11), five contained low DNA concentrations (<0.5 ng/mL), which may have resulted in false negatives. To indicate successful viral amplification, we increased the threshold of Cq values used in a previously published study [18] to 40: however, we classified samples with Cq values of 35 to 40 as having a low viral load. Pox-like lesion tissue samples seem to contain the highest viral load as they have the lowest average Cq value; however, pox-like lesion tissue swabs, remiges, and rectrices also showed significantly low Cq values as well (Table 2). In general, blood samples showed the highest Cq values (Table 2) but amplification of pox virus DNA was successful in all samples (n = 7; Table 1).

**Table 2:** Average ± standard deviation (range) of quantification cycle (Cq) values by sample type for samples taken from Anna’s (n = 26) and *Selasphorus* spp. (n = 1) Hummingbirds and tested via a real-time polymerase chain reaction assay.

Sample Type	Cq Value: Average ± SD (range)
Tissue: Pox-like Lesions	19 ± 5 (14–31)
Tissue: Pectoral Muscle	32 ± 4 (22–38)
Blood	33 ± 3 (29–39)
Toenail Clippings	30 ± 4 (21–39)
Feathers: Rectrices	29 ± 3 (24–37)
Feathers: Remiges	27 ± 3 (25–29)
Feathers: Contour	27 ± 4 (23–38)
Swab (CTA): Pox-like Lesion Tissue	21 ± 4 (16–30)
Swab (CTA): Non Pox-like Lesion Tissue	29 ± 4 (18–36)
Swab (FTA Card): Pox-like Lesion Tissue	22 ± 0 (22–22)

CTA, Cotton-tipped applicator

At the individual bird level, both conventional and real-time PCR assays were able to detect pox virus in at least one sample type for all birds. When explored for various sample types, conventional and real-time PCR assays showed high agreement of 89.72% with a kappa (*k*) of 0.44 (n = 228). Agreement between conventional and real-time PCR assays for various sample types and their corresponding kappa (*k*) values are shown in Table 3. Real-time and conventional PCR results for pox-like lesion swab samples showed perfect agreement in correctly identifying positive samples (n = 42, no *k* was calculated due to lack of observations in category d, Table 3). Feathers as sample types showed moderate concordance when tested with conventional and real-time PCR assays (*k* = 0.449, n = 65 samples).When anatomic location of samples were analyzed, rectrix feathers showed the highest concordance with a *k* of 0.76 (n = 32 samples).

**Table 3:** Summary of the assessment of the performance of a real-time PCR assay for detecting *Avipoxvirus* in samples from Anna’s (n = 26) and *Selasphorus* spp. (n = 1) Hummingbirds. Confusion matrices, Cohen's kappa, positive agreement (PA), and negative agreement (NA) were used to test for agreement between the real-time PCR and conventional PCR assays.

		Agreement with conventional PCR assay						
Sample Type	n (total number of samples)	a	b	c	d	k	PA	NA
Blood	7	6	1	0	0	N/A	0.92	0
Feathers	65	53	8	0	4	0.449	0.92	0.5
Toenail Clippings	29	25	2	1	1	0.345	0.94	0.4
Pox-like Lesion FTA Swab	1	1	0	0	0	N/A	1	N/A
Pox-like Lesion Tissue	43	41	0	2	0	N/A	0.97	0
Muscle	26	23	3	0	0	N/A	0.93	0
Pox-like Lesion Tissue Swab	42	42	0	0	0	N/A	1	N/A
Non Pox-like Lesion Swab	15	12	0	0	3	1	1	1
*Sample types with anatomic location*								
*Feathers*								
Rectrix	32	26	2	0	4	0.76	0.96	0.80
Contour	31	25	6	0	0	N/A	0.89	0
Remige	2	2	0	0	0	N/A	1	N/A
*Toenail Clippings*								
Toenail Clippings	29	25	2	1	1	0.345	0.94	0.40
*Tissue*								
Muscle Pectoral	26	23	3	0	0	0	0.93	0
*Blood*								
Blood	7	6	1	0	0	0	0.92	0
*Pox-like Lesion Tissues*								
Pox-like Lesion Tissue Foot	18	18	0	0	0	N/A	1	N/A
Pox-like Lesion Tissue Beak	14	13	0	1	1	0	0.96	0
Pox-like Lesion Tissue Wing	6	5	0	1	0	0	0.90	0
Pox-like Lesion Tissue Periorbital	3	3	0	0	0	N/A	1	N/A
Pox-like Lesion Tissue Keel	2	2	0	0	0	N/A	1	N/A
*Pox-like Lesion Swabs*								
Pox-like Lesion Swab Wing	7	7	0	0	0	N/A	1	N/A
Pox-like Lesion Swab Beak	16	16	0	0	0	N/A	1	1
Pox-like Lesion Swab Foot	15	15	0	0	0	N/A	1	N/A
Pox-like Lesion Swab Keel	1	1	0	0	0	N/A	1	N/A
Pox-like Lesion Swab Periorbital	3	3	0	0	0	N/A	1	N/A
*Non Pox-like Lesion Swabs*								
Non Pox-like Lesion Swab Beak	6	5	0	0	1	1	1	1
Non Pox-like Lesion Swab Foot	5	5	0	0	0	N/A	1	N/A
Non Pox-like Lesion Swab Periorbital	3	2	0	0	1	1	1	1
Non Pox-like Lesion Swab Wing	1	1	0	0	0	N/A	N/A	1
*Other*								
Pox-like Lesion FTA Swab Foot	1	1	0	0	0	N/A	1	N/A
*Pox-like Lesion vs Pox-like Lesion Swab*								
Pox-like Lesion Tissue	43	41	0	2	0	0	0.97	0
Pox-like Lesion Swab	42	42	0	0	0	N/A	1	N/A
Non Pox-like Lesion Swab	15	12	0	0	3	1	1	1

a: real-time PCR = positive, conventional PCR = positive; b: real-time PCR = positive, conventional PCR = negative; c: real-time PCR = positive, conventional PCR = positive; d: real-time PCR = negative, conventional PCR = positive

**Fig 4:**
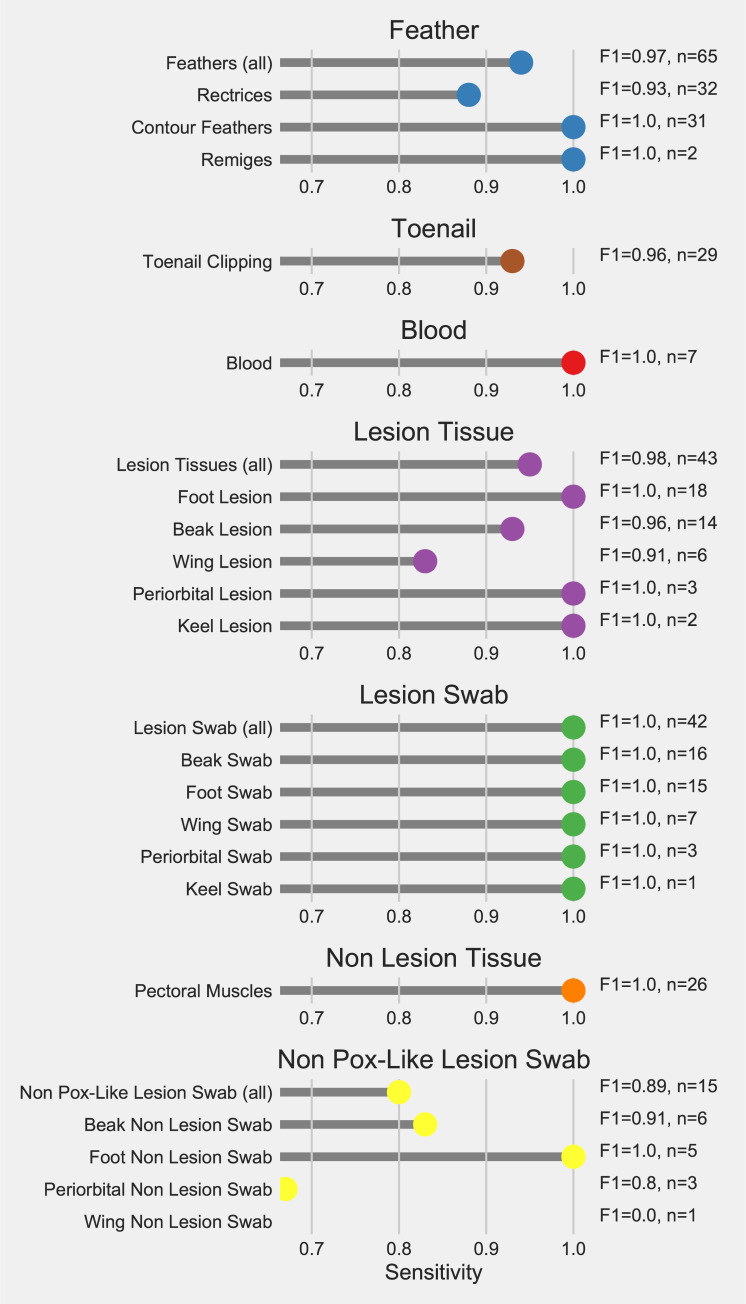
Assessment of performance of a real-time PCR assay for detecting *Avipoxvirus* in samples from Anna’s (n = 26) and *Selasphorus* spp. (n = 1) Hummingbirds in comparison to true infection status. Dots represent the sensitivity of the real-time PCR. F1 score statistics and the number of samples for each sample type are reported next to each sample type. Positive predictive values (PPV) for all sample types were calculated to be 1, except for the pox-like lesion swab of the wing (PPV = 0).

The real-time PCR results showed a high positive predictive value of 1 for all sample types (except non pox-like lesion swabs of wing tissue), while the sensitivity (except for non pox-like lesion swabs) ranged from 0.83 to 1.0 (Fig 4). The values varied within sample type when anatomic region was included in the analysis (Fig 4). Pox-like lesion tissue from the foot, feather, and pectoral muscle tissue samples showed high sensitivity while the sensitivity of real-time PCR was lower for pox-like lesion tissue samples of the beak and wing (Fig 4).

## Discussion

Our results indicate that avian pox can be diagnosed without relying solely on lesion tissue biopsies. Using a variety of integumentary system sample types, pox infections were diagnosed when both conventional and real-time PCR was used. Contour feathers exceled in diagnosing pox viral infections compared to all of the other sample types. To our knowledge, this is the first time that feather samples have been used to diagnose avian pox infections in any bird species. Likewise, we successfully amplified *Avipoxvirus* DNA from swabs taken of pox-like lesions. Our results support that pox viral infections in hummingbirds can be diagnosed with minimally invasive sampling, eliminating the need for anesthesia and reducing risks associated with obtaining tissue biopsies from birds suspected of having pox viral infections.

In this study, we collected and tested multiple sample types to determine which were optimal for diagnosing pox infection. Pox-like lesion tissue was tested as it is the most likely to have high viral load and could be used as a proxy for a “gold standard” for pox detection. Swabs of tissues with and without pox-like lesions were taken and tested to determine if virus could be detected on integument surface areas. Feathers and toenail clip samples were chosen because pox virus targets the integumentary system [9,24,25]. Contour feathers were collected as they are easy to sample and have minimal impact if sampling is conservative. Contour feathers were also chosen as a sample type to compare to remiges and rectrices, which are not optimal for sampling, especially during the breeding season given the importance of rectrices during courtship [26]. Toenail clippings were tested to optimize ante-mortem diagnosis since toenail clipping is a method for obtaining avian blood samples in the field. Whole blood was taken because this is a sample type that can be taken from live birds. Previous studies have found that blood samples may not be reliable for diagnosing avian pox; however, this could be due to low concentrations of circulating virus at the time of sampling a suspected bird [18]. Pectoral muscle tissue was chosen as a sample type, as it can easily be sampled from a carcass intended for a museum collection study skin with minimal damage to the specimen. All tissue (lesion and pectoral muscle), lesion swab, toenail clip, blood and feather samples showed reasonably high sensitivity and could be considered when testing birds for pox infections.

There are several sample types that can be taken from live birds: blood, toenail clippings, contour feathers, remiges, rectrices, and swabs. However, of these sample types, only a few have minimal impact on the bird. As mentioned before, remiges and rectrices are both not optimal for sampling due to potential impacts on flight or breeding success of individual birds. Swabs have potential; however, tissue swabs were most likely to be positive when pox-like lesions were present and non pox-like lesion swabs did not have a high likelihood being positive even when taken from birds that were confirmed positive for infection. There were not enough blood samples tested in this study to determine if it is an optimal sample type for testing. Toenail clippings had a large sample size (>n = 20) and were also highly likely to be positive; however, contour feathers had both a large sample size as well as the highest likelihood of being positive amongst the various sample types that could be taken from live birds. Thus, from our results, we concluded that the best ante-mortem sample type to determine the likelihood of a pox infection are contour feathers. Since contour feathers can be taken, regardless if pox-like lesions are present or not, it is a sample type that can also be used for studying asymptomatic birds.

We tested all samples using both conventional and real-time PCR to determine if there were differences in sensitivity of detecting *Avipoxvirus* DNA. Some samples that were determined negative using conventional PCR were positive when analyzed using real-time PCR testing and returned relatively low Cq values. This finding suggests that real-time PCR has the advantage over conventional PCR in its ability to detect *Avipoxvirus* DNA in samples with low viral load. Specifically, for contour feather samples, real-time PCR yielded a positive result for all birds whereas conventional PCR only detected 77% of the cases. This difference in ability to detect small viral concentrations in samples should also be taken in account when interpreting the predictive values and sensitivity.

Conventional PCR has been used to detect avian pox in the past [2,4] as well as to determine prevalence and genetic diversity of avian pox [27,28]; however, there are still limitations. In analyses using solely conventional PCR, parasite or viral load is difficult to determine [29] and as it relies on downstream visualization, products are handled more than once thus increasing the risk of viral contamination [30]. In addition, compared to real-time PCR, conventional PCR might not be as sensitive for detecting low viral loads. Real-time PCR can be used to determine relative viral load. However, there are also limitations as in some cases, real-time PCR may not detect *Avipoxvirus* DNA in samples taken from pox-positive birds [18]. This was evident in our study, as there were some instances where real-time PCR did not detect avian pox in samples, even those that were found positive using conventional PCR. Despite the limitations, the real-time PCR assay we developed was able to detect very low viral loads (Cq values above 35). Based on our results, real-time PCR holds promise for identifying hummingbirds with pox viral infections using samples, such as feathers, that are taken less invasively. These findings also suggest that using contour feathers as a sample type, asymptomatic birds could also be screened for pox viral infections.

For this study, all sampled hummingbirds had suspected pox lesions on one or more integumentary regions. Since many samples came from carcasses that were wrapped in paper and individually stored in its own bag, there is the possibility of contamination across samples taken from the same individual due to viral particles that may have been dislodged during storage. Therefore, we attempted to address this problem by taking ante-mortem samples to compare to post-mortem samples from the same individual (S1 and S3 Tables). Our results showed that taking samples ante-mortem had just as much success in diagnosing an infected bird as those taken post-mortem thus making sample contamination post-mortem unlikely.

Despite not performing absolute quantification of AAPV DNA in the samples from this study, we developed a protocol for absolute quantification of AAPV DNA that could be used in future studies. Since the main goal for this study was to determine if avian pox infection could be detected without relying on tissue biopsies, we used relative quantification based on Cq values to determine the presence (positive) or absence (negative) of AAPV DNA in all sample types. In addition, there was such a wide range in sample types and lesions, therefore it was challenging to have homogenous sample masses and be able to standardize per sample type. However, in the future it might be a useful tool for pox virus screening, therefore we reported the protocol in this study.

Although hummingbirds are organisms of public interest, there is little known about how avian pox affects hummingbird populations. Hummingbirds are found throughout most of California and the Anna’s Hummingbird in particular, is found in most regions year-round. Hummingbirds can be found in both remote and human-populated areas. With this research, we have expanded our scientific knowledge and determined that contour feathers are an optimal sample type to screen hummingbirds for pox infections without having to rely on tissue biopsies. With the developed assay, we can begin to answer questions regarding *Avipoxvirus* prevalence in wild hummingbird populations, including how human activities may impact the transmission of this disease. As this assay was developed specifically for hummingbirds, it could also provide a means to help determine the transmission routes of the virus: for example, if the virus is spread by insect vectors or through direct contact transmission.

## Supporting information

S1 Table Summary of samples (n = 228 samples) taken from Anna’s (n = 26 birds) and *Selasphorus* spp. (n = 1 bird) Hummingbirds ante-mortem and post-mortem per sample type for testing for *Avipoxvirus*. (DOCX)

S2 Table Summary of results of conventional PCR testing for *Avipoxvirus* for all samples taken from individual hummingbirds (n = 26 Anna’s Hummingbirds and n = 1 *Selasphorus* spp.). (DOCX)

S3 Table Summary of comparison of results of conventional and real-time PCR testing for *Avipoxvirus* for hummingbirds (n = 6) where the same sample types were taken ante-mortem and post-mortem. (DOCX)

S4 Table Summary of Cq value results for real-time PCR testing for *Avipoxvirus* for all samples taken from individual hummingbirds (n = 26 Anna’s Hummingbirds and n = 1 *Selasphorus* spp.). (DOCX)

## References

[1] Bolte AL, Meurer J, Kaleta EF. Avian host spectrum of avipoxviruses. Avian Pathol. 1999 10;28(5):415–432. 10.1080/03079459994434 26911595

[2] Lee LH, Lee KH. Application of the polymerase chain reaction for the diagnosis of fowl poxvirus infection. J Virol Methods. 1997;63(1):113–9. 9015281 10.1016/s0166-0934(96)02119-2

[3] Weli SC, Tryland M. Avipoxviruses: infection biology and their use as vaccine vectors. Virol J. 2011;8:49 10.1186/1743-422X-8-49 21291547 PMC3042955

[4] Godoy LA, Dalbeck LS, Tell LA, Woods LW, Colwell RR, Robinson B, et al Characterization of avian poxvirus in Anna’s hummingbird (Calypte anna) in California, USA. J Wildl Dis. 2013;49(4):978–85. 10.7589/2012-09-230 24502725

[5] Godoy LA, Tell LA, Ernest HB. Hummingbird health: pathogens and disease conditions in the family Trochilidae. J Ornithol. 2014;155(1):1–12.

[6] Fitzner RE, Miller RA, Pierce CA, Rowe SE. Avian pox in a Red-tailed Hawk (Buteo jamaicensis). J Wildl Dis. 1985;21(3):298–301. 10.7589/0090-3558-21.3.298 2993682

[7] Eaves G, Flewett TH. The structure of fowl-pox inclusions (Bollinger bodies). J Hyg (Lond). 1955;53(1):102–5. 14367806 10.1017/s0022172400000541 PMC2217804

[8] Moayyedian H, Mirmohammad-Sadeghi A, Hasanshahi R. Clinical and histopathological survey of lesions similar to pox skin lesions in three flocks of a large commercial layer farm. J Appl Poult Res. 2008;17:556–8.

[9] Boosinger TR, Winterfield RW, Feldman DS, Dhillon AS. Psittacine pox virus: virus isolation and identification, transmission, and cross-challenge studies in parrots and chickens. Avian Dis. 1982;26(2):437–44. 6285884

[10] Feldman RA, Freed LA, Cann RL. A PCR test for avian malaria in Hawaiian birds. Mol Ecol. 1995 12;4(6):663–73. 10.1111/j.1365-294x.1995.tb00267.x 8564006

[11] Waldenström J, Bensch S, Hasselquist D, Östman Ö. A new nested polymerase chain reaction method very efficient in detecting Plasmodium and Haemoproteus infections from avian blood. J Parasitol. 2004;90(1):191–4. 10.1645/GE-3221RN 15040694

[12] Valkiūnas G, Iezhova TA, Križanauskienė A, Palinauskas V, Sehgal RNM, Bensch S. A comparative analysis of microscopy and PCR-based detection methods for blood parasites. J Parasitol. 2008 12;94(6):1395–401. 10.1645/GE-1570.1 18576856

[13] Falcone E, D’Amore E, Di Trani L, Sili A, Tollis M. Rapid diagnosis of avian infectious bronchitis virus by the polymerase chain reaction. J Virol Methods. 1997;64(2):125–30. 10.1016/s0166-0934(96)02151-9 PMC7119601 9079758

[14] Greenwood AG, Blakemore WF. Pox infection in falcons. Vet Rec. 1973;93(17):468–70. 10.1136/vr.93.17.468 4131380

[15] Deern SL, Heard DJ, Fox JH. Avian pox in eastern screech owls and barred owls from Florida. J Wildl Dis. 1997 4;33(2):323–7. 10.7589/0090-3558-33.2.323 9131568

[16] Pérez-Tris J, Williams RAJ, Abel-Fernández E, Barreiro J, Conesa JJ, Figuerola J, et al A multiplex PCR for detection of poxvirus and papillomavirus in cutaneous warts from live birds and museum skins. Avian Dis. 2011 12;55(4):545–53. 10.1637/9685-021411-Reg.1 22312972

[17] Williams RAJ, Escudero Duch C, Pérez-Tris J, Benítez L. Polymerase chain reaction detection of avipox and avian papillomavirus in naturally infected wild birds: comparisons of blood, swab and tissue samples. Avian Pathol. 2014;43(2):130–4. 10.1080/03079457.2014.886326 24456300

[18] Farias MEM, LaPointe DA, Atkinson CT, Czerwonka C, Shrestha R, Jarvi SI. Taqman real-time PCR detects Avipoxvirus DNA in blood of Hawaìi `Amakihi (Hemignathus virens). PLoS One. 2010;5(5):1–6. 10.1371/journal.pone.0010745 PMC2877708 20523726

[19] Jarvi SI, Triglia D, Giannoulis A, Farias M, Bianchi K, Atkinson CT. Diversity, origins and virulence of Avipoxviruses in Hawaiian forest birds. Conserv Genet. 2008;9:339–48.

[20] Weli SC, Traavik T, Tryland M, Coucheron DH, Nilssen Ø. Analysis and comparison of the 4b core protein gene of avipoxviruses from wild birds: Evidence for interspecies spatial phylogenetic variation. Arch Virol. 2004 10;149(10):2035–46. 10.1007/s00705-004-0357-0 15290371

[21] Smith LM, Burgoyne LA. Collecting, archiving and processing DNA from wildlife samples using FTA® databasing paper. BMC Ecol. 2004;4(1):4. 15072582 10.1186/1472-6785-4-4 PMC406513

[22] Cicchetti DV, Feinstein AR. High agreement but low kappa: II. Resolving the paradoxes. J Clin Epidemiol. 1990;43(6):551–8. 10.1016/0895-4356(90)90159-m 2189948

[23] Greiner M, Gardner IA. Epidemiologic issues in the validation of veterinary diagnostic tests. Prev Vet Med. 2000 5;45:3–22. 10.1016/s0167-5877(00)00114-8 10802331

[24] Yoshikawa MGT, Alam J. Histopathological studies of fowl pox in bantams. Int J Poult Sci. 2002;1(6):197–9.

[25] Alehegn E, Chanie M, Mengesha D. A systematic review of serological and clinicopathological features and associated risk factors of avian pox. Brit J Poult Sci. 2014;3(3):78–87.

[26] Clark CJ, Feo TJ. The Anna’s hummingbird chirps with its tail: a new mechanism of sonation in birds. Proc Biol Sci. 2008 4;275(1637):955–62. 10.1098/rspb.2007.1619 18230592 PMC2599939

[27] Gyuranecz M, Foster JT, Dán Á, Ip HS, Egstad KF, Parker PG, et al Worldwide phylogenetic relationship of avian poxviruses. J Virol. 2013 5;87(9):4938–51. 10.1128/JVI.03183-12 23408635 PMC3624294

[28] Ruiz-Martínez J, Ferraguti M, Figuerola J, Martínez-de la Puente J, Williams RAJ, Herrera-Dueñas A, et al Prevalence and genetic diversity of Avipoxvirus in house sparrows in Spain. PLoS One. 2016;11(12):e0168690 10.1371/journal.pone.0168690 28005936 PMC5179100

[29] Nadkarni MA, Martin FE, Jacques NA, Hunter N. Determination of bacterial load by real-time PCR using a broad-range (universal) probe and primers set. Microbiology. 2002 1;148(1): 257–66. 11782518 10.1099/00221287-148-1-257

[30] Heid CA, Stevens J, Livak KJ, Williams PM. Real time quantitative PCR. Genome Res. 1996 10;6(10):986–94. 10.1101/gr.6.10.986 8908518

