# Supplementary material for: TaqMan quantitative real-time PCR for detecting Avipoxvirus DNA in various sample types from hummingbirds
Source: PLoS One. 2020 Jun 11;15(6):e0230701. doi: 10.1371/journal.pone.0230701 (PMC7289624; doi:10.1371/journal.pone.0230701)
Supplement: S1 Table — (DOCX) [file pone.0230701.s001.docx]

**S1 Table. Summary of samples (n=228 samples) taken from Anna’s (n=26 birds) and *Selasphorus* spp. (n=1 bird) Hummingbirds ante-mortem and post-mortem per sample type for testing for *Avipoxvirus*.**

| Sample Type | Ante-Mortem (n=Number of Birds Sampled) | Post-Mortem (n=Number of Birds Sampled) |
| --- | --- | --- |
| Tissue: Pox-like Lesions | 2 (n=2) | 41 (n=23) |
| Tissue: Pectoral Muscle | - | 26 (n=26) |
| Blood | 7 (n=7) | - |
| Toenail Clippings | 5 (n=5) | 24 (n=24) |
| Feathers: Rectrices | 6 (n=6) | 26 (n=26) |
| Feathers: Remiges | 1 (n=1) | 1 (n=1) |
| Feathers: Contour | 4 (n=4) | 27 (n=26) |
| Swab (CTA): Pox-like Lesion Tissue | - | 42 (n=23) |
| Swab (CTA): Non Pox-like Lesion Tissue | - | 15 (n=10) |
| Swab (FTA Card): Pox-like Lesion Tissue | 1 (n=1) | - |
| Total | n=26 | n=202 |

CTA: Cotton-tipped applicator; FTA Card: Whatman FTA Card (GE Healthcare, Chicago, Illinois, USA)
