# Supplementary material for: TaqMan quantitative real-time PCR for detecting Avipoxvirus DNA in various sample types from hummingbirds
Source: PLoS One. 2020 Jun 11;15(6):e0230701. doi: 10.1371/journal.pone.0230701 (PMC7289624; doi:10.1371/journal.pone.0230701)
Supplement: S2 Table — (DOCX) [file pone.0230701.s002.docx]

**S2 Table. Summary of results of conventional PCR testing for *Avipoxvirus* for all samples taken from individual hummingbirds (n=26 Anna’s Hummingbirds and n=1 *Selasphorus* spp.).**

| Bird Number | Tissue: Pox-like Lesion | | Tissue: Pectoral Muscle | Blood | Toenail Clippings | | Feathers: Rectrix | | Feathers: Contour | | Swab (CTA): Pox-like Lesion Tissue | | Swab (CTA): Non Pox-like Lesion Tissue | |
| --- | --- | --- | --- | --- | --- | --- | --- | --- | --- | --- | --- | --- | --- | --- |
| 1^a^ | n/a | | n/a | positive | positive | | n/a | | positive | | n/a | | n/a | |
| 2^bc^ | beak | positive | positive | positive | ante | positive | ante | positive | positive | | beak | positive | periorbital | positive |
|  | wing | positive |  |  | post | positive | post | positive |  |  | wing | positive |  |  |
|  | foot | positive |  |  |  |  |  |  |  |  | foot | positive |  |  |
| 3 | wing | positive | positive | positive | ante | positive | ante | positive | positive | | wing | positive | n/a | |
|  | foot | positive |  |  | post | positive | post | positive |  |  | foot | positive |  |  |
|  | keel | positive |  |  |  |  |  |  |  |  |  |  |  |  |
| 4 | beak | positive | positive | positive | ante | positive | ante | positive | ante | positive | beak | positive | n/a | |
|  | foot | positive |  |  | post | positive | post | positive | post | negative | foot | positive |  |  |
| 5 | foot | positive | positive | positive | ante | positive | ante | positive | ante | positive | foot | positive | n/a | |
|  |  |  |  |  | post | positive | post | positive | post | positive | beak | positive |  |  |
|  |  |  |  |  |  |  |  |  |  |  | wing | positive |  |  |
| 6 | beak | positive | positive | negative | positive | | ante | positive | negative | | beak | positive | n/a | |
|  | foot (ante) | positive |  |  |  |  | post | positive |  |  | foot | positive |  |  |
|  | foot (post) | positive |  |  |  |  |  |  |  |  |  |  |  |  |
| 7 | wing | positive | positive | positive | positive | | positive | | positive | | wing | positive | beak | positive |
| 8 | beak | positive | positive | n/a | positive | | ante | negative | ante | negative | beak | positive | foot | positive |
|  |  |  |  |  |  |  | post | positive | post | positive |  |  |  |  |
| 9 | foot | positive | positive | n/a | positive | | positive | | positive | | foot | positive | n/a | |
|  | periorbital | positive |  |  |  |  |  |  |  |  | periorbital | positive |  |  |
|  | beak | positive |  |  |  |  |  |  |  |  | beak | positive |  |  |
| 10 | beak | positive | positive | n/a | positive | | positive | | positive | | beak | positive | foot | positive |
| 11 | beak | positive | positive | n/a | positive | | positive | | positive | | beak | positive | n/a | |
|  | foot | positive |  |  |  |  |  |  |  |  |  |  |  |  |
| 12 | foot | positive | positive | n/a | positive | | positive | | positive | | foot | positive | n/a | |
|  | beak | positive |  |  |  |  |  |  |  |  | beak | positive |  |  |
|  | wing | positive |  |  |  |  |  |  |  |  | wing | positive |  |  |
| 13 | foot | positive | positive | n/a | positive | | positive | | negative | | foot | positive | n/a | |
|  | beak | positive |  |  |  |  |  |  |  |  | beak | positive |  |  |
| 14 | periorbital | positive | negative | n/a | positive | | positive | | positive | | periorbital | positive | beak | positive |
| 15 | beak | positive | negative | n/a | positive | | negative | | positive | | beak | positive | n/a | |
| 16^b^ | n/a | | positive | n/a | n/a | | positive | | positive | | n/a | | n/a | |
| 17 | n/a | | negative | n/a | negative | | negative | | negative | | n/a | | foot | positive |
|  |  |  |  |  |  |  |  |  |  |  |  |  | beak | positive |
| 18 | beak | positive | positive | n/a | positive | | negative | | positive | | beak | positive | foot | positive |
| 19 | foot | positive | positive | n/a | negative | | negative | | positive | | foot | positive | beak | negative |
|  |  |  |  |  |  |  |  |  |  |  |  |  | wing | negative |
|  |  |  |  |  |  |  |  |  |  |  |  |  | periorbital | negative |
| 20 | beak | positive | positive | n/a | positive | | positive | | positive | | beak | positive | n/a | |
|  | foot | positive |  |  |  |  |  |  |  |  |  |  |  |  |
| 21^d^ | foot | positive | positive | n/a | n/a | | positive | | 1 | positive | foot | positive | n/a | |
|  |  |  |  |  |  |  |  |  | 2 | positive |  |  |  |  |
| 22 | n/a | | positive | n/a | negative | | negative | | negative | | n/a | | beak | positive |
|  |  |  |  |  |  |  |  |  |  |  |  |  | foot | positive |
| 23 | foot | positive | positive | n/a | positive | | positive | | positive | | foot | positive | n/a | |
|  |  |  |  |  |  |  |  |  |  |  | beak | positive |  |  |
| 24 | wing | positive | positive | n/a | positive | | positive | | positive | | wing | positive | n/a | |
|  | foot | positive |  |  |  |  |  |  |  |  | foot | positive |  |  |
| 25 | periorbital | positive | positive | n/a | positive | | positive | | positive | | periorbital | positive | n/a | |
|  | foot | positive |  |  |  |  |  |  |  |  | foot | positive |  |  |
|  | beak | positive |  |  |  |  |  |  |  |  | beak | positive |  |  |
| 26 | foot | positive | positive | n/a | positive | | positive | | positive | | foot | positive | periorbital | positive |
|  | keel | positive |  |  |  |  |  |  |  |  | keel | positive | beak | positive |
|  | wing | positive |  |  |  |  |  |  |  |  | wing | positive |  |  |
| 27 | foot | positive | positive | n/a | positive | | positive | | positive | | foot | positive | n/a | |
|  | beak | positive |  |  |  |  |  |  |  |  | beak | positive |  |  |

CTA: Cotton-tipped applicator; n/a: No sample of this sample type was taken.

^a^ All samples from this bird were taken ante-mortem.

^b^ One remige/bird was sampled and tested from birds 2 and 16; both remige samples tested positive via conventional PCR.

^c^ A swab was taken of tissue that had a pox-like lesion using an FTA card (Whatman FTA card, GE Healthcare, Chicago, Illinois, USA); this FTA swab sample tested positive via conventional PCR.

^d^ For this bird, two sets of post-mortem contour feather samples were taken and tested.
