# Supplementary material for: TaqMan quantitative real-time PCR for detecting Avipoxvirus DNA in various sample types from hummingbirds
Source: PLoS One. 2020 Jun 11;15(6):e0230701. doi: 10.1371/journal.pone.0230701 (PMC7289624; doi:10.1371/journal.pone.0230701)
Supplement: S3 Table — (DOCX) [file pone.0230701.s003.docx]

**S3 Table. Summary of comparison of results of conventional and real-time PCR testing for *Avipoxvirus* for hummingbirds (n=6) where the same sample types were taken ante-mortem and post-mortem.**

| Bird Number | Sample Type | Ante-Mortem Results (Conventional PCR) | Post-Mortem Results (Conventional PCR) | Ante-Mortem Results (Real-time PCR; Cq Value) | Post-Mortem Results (Real-time PCR; Cq Value) |
| --- | --- | --- | --- | --- | --- |
| 2 | Rectrix | Positive | Positive | 30.09 | 24.18 |
| 2 | Toenail Clipping | Positive | Positive | 32.7 | 32.13 |
| 3 | Rectrix | Positive | Positive | 28.04 | 27.73 |
| 3 | Toenail Clipping | Positive | Positive | 28.61 | 26.5 |
| 4 | Rectrix | Positive | Positive | 30.00 | 28.95 |
| 4 | Toenail Clipping | Positive | Positive | 28.93 | 30.79 |
| 4 | Contour Feathers | Positive | Negative | 27.04 | 25.09, 25.15 |
| 5 | Rectrix | Positive | Positive | 32.21 | 30.22 |
| 5 | Toenail Clipping | Positive | Positive | 26.77 | 20.70 |
| 5 | Contour Feathers | Positive | Positive | 28.29 | 27.69 |
| 6 | Rectrix | Positive | Positive | 29.04 | 29.30 |
| 6 | Pox-like Lesion Tissue | Positive | Positive | 16.08 | 15.58 |
| 8 | Rectrix | Negative | Positive | n/a | 29.78 |
| 8 | Contour Feathers | Negative | Positive | 37.40 | 24.39 |

Cq: Cycle quantification; Cq values of 40 and below were considered to indicate successful viral amplification but Cq values of 35-40 were considered as being indicative of low viral load; n/a: This sample was considered to be negative for *Avipoxvirus* when run through real-time PCR.
