# Supplementary material for: TaqMan quantitative real-time PCR for detecting Avipoxvirus DNA in various sample types from hummingbirds
Source: PLoS One. 2020 Jun 11;15(6):e0230701. doi: 10.1371/journal.pone.0230701 (PMC7289624; doi:10.1371/journal.pone.0230701)
Supplement: S4 Table — (DOCX) [file pone.0230701.s004.docx]

**S4 Table. Summary of Cq value results for real-time PCR testing for *Avipoxvirus* for all samples taken from individual hummingbirds (n=26 Anna’s Hummingbirds and n=1 *Selasphorus* spp.).**

| Bird Number | Tissue: Pox-like Lesion | | Tissue: Pectoral Muscle | Blood | Toenail Clippings | | Feathers: Rectrix | | Feathers: Contour | | Swab (CTA): Pox-like Lesion Tissue | | Swab (CTA): Non Pox-like Lesion Tissue | |
| --- | --- | --- | --- | --- | --- | --- | --- | --- | --- | --- | --- | --- | --- | --- |
| 1^a^ | n/a | | n/a | 30.75 | 27.14 | | n/a | | 28.26 | | n/a | | n/a | |
| 2^bc^ | beak | 17.7 | 28.4 | 30.17 | ante | 32.7 | ante | 30.09 | 24.72 | | beak | 21 | periorbital | 18.49 |
|  | wing | negative |  |  | post | 32.13 | post | 24.18 |  |  | wing | 18.7 |  |  |
|  | foot | 17.07 |  |  |  |  |  |  |  |  | foot | 16.21 |  |  |
| 3 | wing | 20.1 | 29.94 | 32.77 | ante | 28.61 | ante | 28.04 | 26.19 | | wing | 19.43 | n/a | |
|  | foot | 21.34 |  |  | post | 26.5 | post | 27.73 |  |  | foot | 15.72 |  |  |
|  | keel | 28.44 |  |  |  |  |  |  |  |  |  |  |  |  |
| 4 | beak | 16.03 | 29.64 | 35.34 | ante | 28.93 | ante | 30 | ante | 27.04 | beak | 21.72 | n/a | |
|  | foot | 17.32 |  |  | post | 30.79 | post | 28.95 | post | 25.09, 25.15 | foot | 19.08 |  |  |
| 5 | foot | 19.22 | 30.15 | 35.05 | ante | 26.77 | ante | 32.21 | ante | 28.29 | foot | 19.4 | n/a | |
|  |  |  |  |  | post | 20.7 | post | 30.22 | post | 27.69 | beak | 18.72 |  |  |
|  |  |  |  |  |  |  |  |  |  |  | wing | 27.84 |  |  |
| 6 | beak | 15.63 | 29.09 | 38.64 | 28.51 | | ante | 29.04 | 24.88, 25.23 | | beak | 21.56 | n/a | |
|  | foot (ante) | 16.08 |  |  |  |  | post | 29.3 |  |  | foot | 16.74 |  |  |
|  | foot (post) | 15.58 |  |  |  |  |  |  |  |  |  |  |  |  |
| 7 | wing | 16.72 | 33.79 | 29.26 | 32.41 | | 29.93 | | 29.77 | | wing | 23.78 | beak | 32.49 |
| 8 | beak | 22.05 | 29.44 | n/a | 24.97 | | ante | negative | ante | 37.4 | beak | 21.05 | foot | 25.05 |
|  |  |  |  |  |  |  | post | 29.78 | post | 24.39 |  |  |  |  |
| 9 | foot | 19.69 | 31.64 | n/a | 29.11 | | 28.2 | | 27.34 | | foot | 24.09 | n/a | |
|  | periorbital | 16.87 |  |  |  |  |  |  |  |  | periorbital | 20.11 |  |  |
|  | beak | 15.03 |  |  |  |  |  |  |  |  | beak | 25.21 |  |  |
| 10 | beak | 17.65 | 27.99 | n/a | 28.13 | | 27.6 | | 26.67 | | beak | 20.37 | foot | 27.7 |
| 11 | beak | 18.83 | 35.69 | n/a | 33.49 | | 28.83 | | 24.11 | | beak | 20.63 | n/a | |
|  | foot | 16.07 |  |  |  |  |  |  |  |  |  |  |  |  |
| 12 | foot | 14.50, 14.74, 15.38 | 35.43 | n/a | 29.56 | | 28.79 | | 25.62 | | foot | 20.87 | n/a | |
|  | beak | 15.94 |  |  |  |  |  |  |  |  | beak | 24.76 |  |  |
|  | wing | 17.67 |  |  |  |  |  |  |  |  | wing | 23.64 |  |  |
| 13 | foot | 18.21 | 31.05 | n/a | 31.82 | | 28.35 | | 27.09, 27.4 | | foot | 19.12 | n/a | |
|  | beak | 17.11 |  |  |  |  |  |  |  |  | beak | 18.32 |  |  |
| 14 | periorbital | 17.51 | 36.94 | n/a | 31.01 | | 32.31 | | 26.87 | | periorbital | 17.23 | beak | 29.39 |
| 15 | beak | 18.32 | 37.92 | n/a | 31.03 | | 35.74 | | 28.58 | | beak | 19.65 | n/a | |
| 16^b^ | n/a | | 32.12 | n/a | n/a | | 30.87 | | 23.39 | | n/a | | n/a | |
| 17 | n/a | | 38.4 | n/a | 38.63 | | negative | | 35.64 | | n/a | | foot | 29.42 |
|  |  |  |  |  |  |  |  |  |  |  |  |  | beak | 28.32 |
| 18 | beak | negative | 21.6 | n/a | negative | | negative | | 34.36 | | beak | 29.05 | foot | 28.31 |
| 19 | foot | 29.02 | 32.25 | n/a | negative | | negative | | 36.15 | | foot | 29.63 | beak | negative |
|  |  |  |  |  |  |  |  |  |  |  |  |  | wing | negative |
|  |  |  |  |  |  |  |  |  |  |  |  |  | periorbital | negative |
| 20 | beak | 18.36 | 29.04 | n/a | 31.05 | | 27.28 | | 26.22 | | beak | 18.37 | n/a | |
|  | foot | 31.12 |  |  |  |  |  |  |  |  |  |  |  |  |
| 21^d^ | foot | 16.34 | 30.29 | n/a | n/a | | 24.74 | | 1 | 23.14 | foot | 19.19 | n/a | |
|  |  |  |  |  |  |  |  |  | 2 | 23.37, 24.01 |  |  |  |  |
| 22 | n/a | | 36.14 | n/a | 38.52 | | 37.44 | | 37.79 | | n/a | | beak | 36.08 |
|  |  |  |  |  |  |  |  |  |  |  |  |  | foot | 32.79 |
| 23 | foot | 18.89 | 31.23 | n/a | 27.19 | | 25.25 | | 24.09 | | foot | 18.76 | n/a | |
|  |  |  |  |  |  |  |  |  |  |  | beak | 19.55 |  |  |
| 24 | wing | 29.31 | 31.85 | n/a | 28.36 | | 23.84 | | 25.44 | | wing | 28.01 | n/a | |
|  | foot | 16.15 |  |  |  |  |  |  |  |  | foot | 22.23 |  |  |
| 25 | periorbital | 21.34 | 30.17 | n/a | 25.49 | | 23.59 | | 24.39 | | periorbital | 16.81 | n/a | |
|  | foot | 28.04 |  |  |  |  |  |  |  |  | foot | 22.88 |  |  |
|  | beak | 26.25, 26.03 |  |  |  |  |  |  |  |  | beak | 24.59 |  |  |
| 26 | foot | 18.11 | 32.13 | n/a | 32.86 | | 25.09 | | 24.36 | | foot | 23.32 | periorbital | 31.23 |
|  | keel | 30.02 |  |  |  |  |  |  |  |  | keel | 20.56 | beak | 30.8 |
|  | wing | 18.81 |  |  |  |  |  |  |  |  | wing | 27.46 |  |  |
| 27 | foot | 16.01 | 37.5 | n/a | 27.38 | | 30.48 | | 27.88 | | foot | 19.63 | n/a | |
|  | beak | 17.31 |  |  |  |  |  |  |  |  | beak | 20.36 |  |  |

Cq: Cycle quantification; Cq values of 40 and below were considered to indicate successful viral amplification but Cq values of 35-40 were considered as being indicative of low viral load; CTA: Cotton-tipped applicator; n/a: No sample of this sample type was taken.

^a^ All samples from this bird were taken ante-mortem.

^b^ One remige/bird was sampled and tested from birds 2 and 16; both remiges tested positive via real-time PCR: Bird 2 with a Cq value of 25.24 and Bird 16 with a Cq value of 29.2.

^c^ A swab was taken of tissue that had a pox-like lesion using an FTA card (Whatman FTA card, GE Healthcare, Chicago, Illinois, USA); this FTA swab sample tested positive via real-time PCR with a Cq value of 22.26.

^d^ For this bird, two sets of post-mortem contour feather samples were taken and tested.
